# Reply to: Neurocritical care management supported by multimodal brain monitoring after acute brain injury

**DOI:** 10.62675/2965-2774.20240187-en

**Published:** 2024-09-20

**Authors:** Elisabete Monteiro, Sofia Rocha e Silva, Marek Czosnyka, José Artur Paiva, Celeste Dias

**Affiliations:** 1 Universitário São João Centro Hospitalar Department of Intensive Care Medicine Porto Portugal Department of Intensive Care Medicine, Centro Hospitalar e Universitário São João - Porto, Portugal.; 2 Universidade do Porto Faculdade de Medicina Porto Portugal Faculdade de Medicina, Universidade do Porto - Porto, Portugal.; 3 University of Cambridge Division of Neurosurgery Department of Clinical Neurosciences Cambrigde United Kingdom Brain Physics Laboratory, Division of Neurosurgery, Department of Clinical Neurosciences, University of Cambridge - Cambrigde, United Kingdom.

To the Editor

We read with interest the commentary by Josef Finsterer et al.^(1)^ about our article,^(2)^ and we truly thank the authors for bringing to light for discussion several important issues with implications for daily clinical practice.

We fully agree that there are other factors influencing the outcomes in traumatic brain injury (TBI) and subarachnoid hemorrhage (SAH) patients, but our rationale was to select a population of patients with TBI or SAH severe enough to require admission to a level III intensive care unit (ICU), independent of which specific factors led to that degree of severity. Similarly, we agree that the Simplified Acute Physiology Score (SAPS) II may not be sufficient for assessing TBI or SAH severity, but it is a more global, systemic, and physiological severity score than the Hunt-Hess and Fisher classifications are. These patients are systemically and critically ill and not only severely neurologically injured.

A second limitation refers to the insufficient characterization of Group 1 (G1) and Group 2 (G2). In a previous paper,^(3)^ a comparison was made between TBI patients and SAH patients, confirming our clinical and subjective perceptions that TBI patients have, at baseline, a more severe condition, with higher SAPS II scores and higher incidences of hypoxemia and hypotension. Despite only slight differences between the two groups regarding mortality (except at 6 months, when mortality was higher in TBI patients), SAH patients had better outcomes than did TBI patients. We hypothesized that this may be dependent on the initial insult and the inability to promptly revert causes of secondary lesions, such as hypoperfusion and hypoxemia, since the initial diagnosis (either TBI or SAH) does not limit or direct the type of monitoring given.^(3)^ In G1, 23 patients (33%) had SAH. In G2, 72 patients had SAH (23%).

The third limitation is that the Hunt-Hess score was not reported for SAH patients. We agree that the severity assessment of SAH is more accurate with specific scales, but the Glasgow Coma Scale (GCS) is the scale we consider more correct to use when comparing the neurological status between TBI and SAH patients. In 50% of patients with SAH, the Hunt-Hess score was 4 or 5, and in 95% of patients, the Fisher score was 4, highlighting the severity of the condition of the included patients.

The fourth limitation is the lower mean GCS score and higher SAPS II score in some subsets of patients. The allocation of patients to the general ICU occurred due to a shortage of available beds in the neurocritical care unit, and the decision was made by the intensivist in charge of the emergency room. A bias may exist due to bed selection and availability, since beds could be made available depending on the potential for survival of the patient.

The fifth limitation is the greater likelihood of side effects with more invasive monitoring. Infection is a major concern with external ventricular drainage^(4)^ but not with intracranial pressure, a thermal diffusion probe for cerebral blood flow measurement or oxygen brain tension evaluation. However, external ventricular drainage is not used as a tool for brain monitoring by itself but rather as a treatment for hydrocephalus. With respect to other complications, they are rare.

We believe that these pertinent comments from Finsterer et al. enriched our paper.^(1)^

## References

[1] Finsterer J, Scorza FA (2024). To: Neurocritical care management supported by multimodal brain monitoring after acute brain injury. Crit Care Sci.

[2] Monteiro E, Ferreira A, Mendes ER, Silva SR, Maia I, Dias CC (2023). Neurocritical care management supported by multimodal brain monitoring after acute brain injury. Crit Care Sci.

[3] Monteiro E, Ferreira A, Mendes E, Dias CC, Czosnyka M, Paiva JA, Depreitere B, Meyfroidt G, Güiza F (2021). Intracranial pressure and neuromonitoring XVII.

[4] Cucciolini G, Motroni V, Czosnyka M (2023). Intracranial pressure for clinicians: it is not just a number. J Anesth Analg Crit Care.

