# Supplementary material for: Reply to: Neurocritical care management supported by multimodal brain monitoring after acute brain injury
Source: Crit Care Sci. 2024 Sep 20;36:e20240187en. doi: 10.62675/2965-2774.20240187-en (PMC11463983; doi:10.62675/2965-2774.20240187-en)
Supplement: Translation

# Translation: Resposta para: Abordagem de cuidados neurocríticos apoiada por monitorização cerebral multimodal após lesão cerebral aguda

**DOI:** 10.62675/2965-2774.20240187-pt

**Authors:** Elisabete Monteiro, Sofia Rocha e Silva, Marek Czosnyka, José Artur Paiva, Celeste Dias

**Affiliations:** 1 Porto Portugal Departamento de Medicina Intensiva, Centro Hospitalar e Universitário São João - Porto, Portugal.; 2 Porto Portugal Faculdade de Medicina, Universidade do Porto - Porto, Portugal.; 3 Cambrigde Reino Unido Brain Physics Laboratory, Division of Neurosurgery, Department of Clinical Neurosciences, University of Cambridge - Cambrigde, Reino Unido.

Ao Editor

Lemos com interesse o comentário de Josef Finsterer et al.^(1)^ sobre nosso artigo^(2)^ e agradecemos sinceramente aos autores por trazerem à tona para discussão várias questões importantes com implicações na prática clínica diária.

Concordamos plenamente que há outros fatores que influenciam os desfechos em pacientes com traumatismo craniencefálico (TCE) e hemorragia subaracnoidea (HAS), no entanto, nossa justificativa foi selecionar uma população de pacientes com TCE ou HAS grave o suficiente para exigir admissão numa unidade de terapia intensiva (UTI) nível III, independentemente dos fatores específicos que levaram a esse grau de gravidade. Da mesma forma, concordamos que o *Simplified Acute Physiology Score* (SAPS) II talvez não seja suficiente para avaliar a gravidade do TCE ou da HAS, embora seja um escore de gravidade mais global, sistêmico e fisiológico do que as classificações de Hunt-Hess e Fisher. Esses pacientes estão sistêmica e gravemente comprometidos e não apenas com lesões neurológicas graves.

Uma segunda limitação refere-se à caracterização insuficiente do Grupo 1 (G1) e do Grupo 2 (G2). Em artigo anterior,^(3)^ fizemos uma comparação entre pacientes com TCE e pacientes com HAS, confirmando nossas percepções clínicas e subjetivas de que os pacientes com TCE têm, basalmente, uma condição mais grave, com SAPS II mais altos e maior incidência de hipoxemia e hipotensão. Apesar de haver apenas pequenas diferenças entre os dois grupos com relação à mortalidade (exceto aos 6 meses, quando a mortalidade foi maior nos pacientes com TCE), os pacientes com HAS tiveram desfechos mais favoráveis do que aqueles com TCE. Levantamos a hipótese de que isso pode depender do trauma inicial e da incapacidade de reverter prontamente as causas de lesões secundárias, como hipoperfusão e hipoxemia, uma vez que o diagnóstico inicial (TCE ou HAS) não limita nem direciona o tipo de monitoramento realizado.^(3)^ No G1, 23 pacientes (33%) tinham HAS. No G2, 72 pacientes tinham HAS (23%).

A terceira limitação é que o escore de Hunt-Hess não foi relatado para pacientes com HAS. Concordamos que a avaliação da gravidade da HAS é mais precisa com escalas específicas, mas a escala de coma de Glasgow é a que consideramos mais correta ao comparar o estado neurológico entre pacientes com TCE e HAS. Em 50% dos pacientes com HAS, o escore de Hunt-Hess foi 4 ou 5, e em 95% dos pacientes, o escore de Fisher foi 4, destacando a gravidade da condição dos pacientes incluídos.

A quarta limitação é a escala de coma de Glasgow média mais baixa e o escore SAPS II mais alto em alguns subgrupos de pacientes. A alocação de pacientes na UTI geral ocorreu devido à falta de leitos disponíveis na unidade de cuidados neurocríticos, e a decisão foi tomada pelo intensivista responsável pela emergência. Pode haver um viés devido à seleção e à oferta de leitos, uma vez que os leitos podem ser alocados dependendo do potencial de sobrevida do paciente.

A quinta limitação é a maior probabilidade de efeitos colaterais com o monitoramento mais invasivo. A infecção é uma grande preocupação com a drenagem ventricular externa,^(4)^ mas não com a pressão intracraniana, uma sonda de difusão térmica para medição do fluxo sanguíneo cerebral ou avaliação da tensão cerebral de oxigênio. No entanto, a drenagem ventricular externa não é usada como uma ferramenta de monitoramento cerebral por si só, mas como um tratamento da hidrocefalia. Com relação a outras complicações, elas são raras.

Acreditamos que esses comentários pertinentes de Finsterer et al. enriqueceram nosso artigo.^(1)^
